# Psoriasis and Systemic Inflammatory Disorders

**DOI:** 10.3390/ijms23084457

**Published:** 2022-04-18

**Authors:** Tomoko Tashiro, Yu Sawada

**Affiliations:** Department of Dermatology, University of Occupational and Environmental Health, Kitakyushu 807-8555, Japan; tomoko-t@med.uoeh-u.ac.jp

**Keywords:** psoriasis, IL-17, TNF-α, organs, inflammation

## Abstract

Psoriasis is a representative inflammatory skin disease occupied by large surface involvement. As inflammatory cells and cytokines can systemically circulate in various organs, it has been speculated that psoriatic skin inflammation influences the systemic dysfunction of various organs. Recent updates of clinical studies and experimental studies showed the important interaction of psoriasis to systemic inflammatory diseases. Furthermore, the importance of systemic therapy in severe psoriasis is also highlighted to prevent the development of systemic inflammatory diseases. In this review, we introduced representative systemic inflammatory diseases associated with psoriasis and the detailed molecular mechanisms.

## 1. Introduction

The skin is the outmost layer organ located in the human body and is exposed to various environmental factors, which activate inflammatory skin responses [1,2]. Since cutaneous immune cells and inflammatory cytokines systemically circulate in the whole body [3,4], it is assumed that skin inflammation has the potential to become a trigger to enhance inflamed systemic organ reactions and dysfunction; however, little is known regarding the detailed mechanisms of inflamed skin-induced systemic organ dysfunctions.

Psoriasis is a representative inflammatory skin disease characterized by a scaly erythematous eruption [5]. Histologically, psoriatic skin shows epithelial hyperplasia with parakeratosis along with the infiltration of various inflammatory cells, such as lymphocytes and neutrophils, in the dermis [6], suggesting that an inflammatory response manifests the clinical characteristics of psoriasis. Recent studies identified that psoriatic skin inflammation becomes a source of inflammatory reaction involved in various systemic inflammatory disorders. In psoriasis patients, the risk of cardiovascular disease [7] and bone fracture is especially increased [8]. In addition, psoriatic arthritis (PsA) shows a high frequency of incidence in psoriasis patients and sometimes develops into a severe dysfunction of arthritis [9]; therefore, the presence of these risks and complications should be kept in mind in the clinical scenario. As a representative psoriatic inflammatory disease, PsA is a disease state with joint symptoms in psoriasis [9]. The early detection of the presence of PsA is important to avoid joint deformity or destruction [10]. In PsA, mechanical irritation similar to the Koebner phenomenon of the skin causes inflammation in the attachments. Adhesion inflammation occurs near the DIP joint to accessory ligaments and often inflammation spreads to the nail bed, leading to nail involvement.

Psoriasis causes systemic inflammatory responses and sometimes causes tissue and organ dysfunction [11,12,13,14]. Indeed, several studies were conducted to elucidate the disease’s risk and pathogenesis of the dysfunction. To highlight the whole interaction of psoriatic skin inflammation to systemic organ involvement, this review introduces the detailed risk and molecular mechanisms that enhance the inflammatory reaction mediated by psoriatic skin inflammation.

## 2. The Pathogenesis of Psoriasis

**Figure 1:**
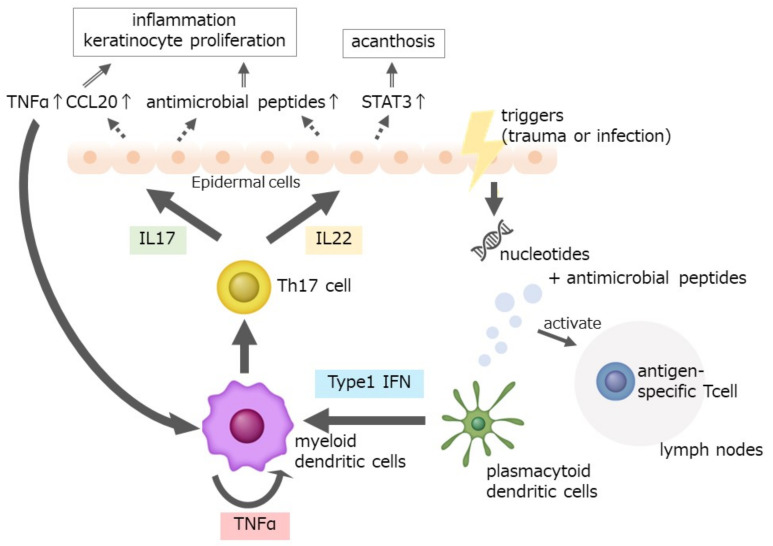
The pathogenesis of psoriasis. External triggers of trauma or infection induce host cell-derived nucleotides, which make a complex with keratinocytes-derived antimicrobial peptides. This complex is recognized by antigen-presenting cells, such as plasmacytoid dendritic cells, and activates antigen-specific T cell expansion in the skin and lymph nodes. Plasmacyte dendritic cell produces type I interferons, which activate the secretion of IL-23 and TNF by myeloid dendritic cells. These cytokines enhance the production of IL-17 and IL-22 by Th17 cells, which are activated by IL-1. IL-17 activates the production of TNF, CCL20, and antimicrobial peptides to enhance the inflammatory reaction in the skin and the proliferation of keratinocytes. The importance of these inflammatory cytokines has been proven by the specific cytokine inhibitors, which show strong anti-inflammatory action against psoriatic skin inflammation.

External triggers of trauma or infection induce host cell-derived nucleotides, which make a complex with keratinocyte-derived antimicrobial peptides [15]. This complex is recognized by antigen-presenting cells, such as plasmacytoid dendritic cells, and activates antigen-specific T cell expansion in the skin and lymph nodes [15]. The plasmacyte dendritic cell produces type I interferons, which activate the secretion of IL-23 and TNF by myeloid dendritic cells [16]. These cytokines enhance the production of IL-17 and IL-22 by Th17 cells, which are activated by IL-1. IL-17 activates the production of TNF, CCL20, and antimicrobial peptides which enhance the inflammatory reaction in the skin and the proliferation of keratinocytes [17,18]. The importance of these inflammatory cytokines has been proven by the specific cytokine inhibitors, which show strong anti-inflammatory action against psoriatic skin inflammation [19]. As environmental factors, diet foods, microorganisms, and their products of fatty acids are also involved in the development of psoriasis [20,21,22,23] (Figure 1).

In addition, these inflammatory cytokines are not limited to skin inflammation, as they also develop into systemic inflammatory diseases; therefore, we next introduce representative systemic inflammatory diseases mediated by psoriatic skin inflammation.

## 3. Cerebrocardiovascular Diseases

Cerebrocardiovascular diseases, especially myocardial infarction and cerebrovascular infarction, are emergency fatal events by the complete blockage of a coronary or cerebrovascular artery caused by a rupture of an atherosclerotic plaque or artery spasms [24]. Although the incidence rate gradually decreased, the incidence of myocardial infarction was still a large population in approximately 550,000 first episodes and 200,000 recurrent episodes in the United States (a large cohort study) [25].

Various studies have investigated the relationship between psoriasis and myocardial infarction or cerebrovascular diseases [26,27,28,29]. Psoriasis increased the risk of myocardial infarction [7] and stroke (Rate ratio = 1.97, mild psoriasis *n* = 36,765) (a large cohort study) [30]. The severity of psoriasis is also an important factor for the development of the risk of cerebrocardiovascular diseases (Rate ratio = 2.80, severe psoriasis *n* = 2793) (a large cohort study) [30]. PASI scores of more than 10 showed higher risks of coronary heart disease (28% greater for coronary heart disease) (a large cohort study) [31]; therefore, severe psoriasis needs to be taken care of in the occurrence and the evaluation of cerebrovascular diseases. A lipid-rich necrotic core (LRNC) is a representative characteristic of high-risk coronary plaque, which is a feature that causes future cardiovascular events. LRNC was associated with the severity of psoriasis and biologic therapy which reduced LRNC development (*n* = 209) (a small prospective study) [32]. On the contrary, overall rates of acute myocardial infarction mortality were significantly lower in PsA patients compared with those without PsA (PsA vs. non-PsA population = 2.21% vs. 5.8%, PsA patient *n* = 4778) (a large cohort study) [33]. Systemic treatment is important to reduce the risk of vascular events. The TNF inhibitor treatment showed lower hazard ratios of myocardial infarction (HR = 0.50, psoriasis patient *n* = 8845) and stroke (incidence rate = 0.30, psoriasis patient *n* = 72,373) (a large cohort study) [34,35]. An IL-17 inhibitor, secukinumab, also showed a reduced aortic vascular inflammation and biomarkers of cardiometabolic disease [36].

Dilated blood vessels and lymphatic vessels were observed in psoriasis lesional skin and exchanged with blood plasma containing psoriasis inflammatory cytokines, which can systemically circulate [17]. Dominant cytokine profiles of IFN-γ and TNF-α were overlapped with psoriasis lesional skin and atherosclerotic plaque which induced the inflammatory reaction in the endothelium [37]. Circulating IL-17 producing cells were identified in circulating peripheral blood and cultured atherosclerotic coronary arteries in patients with coronary atherosclerosis [38]. Epidermal IL-17A overexpression enhanced endothelial dysfunction [39]. Endothelial cells in patients with psoriasis upregulated VCAM-1, IL-1β, and COX-2 [40]. These cytokine-induced endothelial damages accelerated systemic inflammasome signaling induced via TNF-α and IL-17A signaling [41,42]. Platelets contribute to the pathogenesis of vascular dysfunction. Psoriasis platelet elevated expression of COX-1 correlated with psoriasis skin severity [41,43], suggesting a possible beneficial impact of COX-1 inhibitor to prevent the cardiovascular events in psoriasis [44].

## 4. Osteoporosis

Osteoporosis is characterized by low bone mass and deterioration of bone tissue with an increasing bone fragility [45]. Osteoporosis-associated bone fractures become increasingly common in the elderly population [45]. Bone remodeling is a process for the replacement of old bone for the renewal of the skeletons. The recruitment of osteoclasts is followed by resorption of mineralized bone, and osteoblasts are recruited to the site and cause mineralization of new bone.

Several statistical studies investigated the association of psoriasis with osteoporosis and fracture. A cross-sectional study in the United States population showed that psoriasis patients (183,725 with psoriasis and 28,765 with PsA) had a significantly higher ratio of osteoporosis (HR = 2.97, 2.89–3.06) and pathological fractures (HR = 2.35, 2.19–2.53) (a large cohort study) [8]. Another cohort study in the United Kingdom population also showed that psoriasis patients (*n* = 24,219) had a 10% increased risk of fracture (HR = 1.10, 1.04–1.16) (a large cohort study) [46]. The degree of severity of psoriasis skin inflammation tended to increase the risk of fracture. Although patients with mild psoriasis had a slightly elevated risk of fractures (HR = 1.07, 1.05–1.10), patients with severe psoriasis had a highly elevated risk of fractures (HR = 1.26, 1.15–1.39) (PsA, *n* = 9788; psoriasis, *n* = 158,323) (a large cohort study) [47]. These findings indicate a possible therapeutic benefit against psoriasis to prevent the occurrence of fracture or osteoporosis.

TNF-α is involved in the pathogenesis of osteoporosis in psoriasis. TNF-α enhanced bone resorption mediated osteoclast activation [48]. IL-6 also stimulated bone resorption [48]. In addition, IL-23/IL-17 signal pathway also develops osteoporosis. IL-17 plays an important role in the development of osteoclasts. Anti-IL-17 antibodies significantly inhibited osteoclast formation [49]. Furthermore, IL-23 enhances bone loss mediated by increased osteoclastogenesis and the receptor activator of kappa B ligand (RANKL) expression in T cells [50]. IL-23 activated the tartrate-resistant acid phosphatase (TRAP) in osteoclasts, which was essential for the development of osteoporosis [50]. Indeed, the combination of anti-IL-23 and anti-IL17 impaired osteoporosis and prevented bone loss [50]. Although no study has been conducted, it is expected that the efficacy of systemic therapy might be observed in psoriasis to prevent osteoporosis.

## 5. Liver Dysfunction

Nonalcoholic fatty liver disease (NAFLD) is characterized by excess accumulation of fat in hepatocytes, leading to liver dysfunction [51]. In addition, fatty acids have toxicities to hepatocytes following liver-deposited fatty acids-induced cell death, which cause the release of damage-associated molecular patterns (DAMPs) into the extracellular space, which are recognized by recruited macrophages and activates TLRs and inflammasome pathways to enhance the production of pro-inflammatory cytokines, which exacerbates hepatic inflammation [51].

Psoriasis and NAFLD are both inflammatory diseases and statistical analysis showed the relationship between psoriasis and NAFLD. The prevalence of NAFLD was higher in psoriasis patients (44–47% in psoriasis vs. 26–28% in control) (small cohort studies) [52,53]. PsA also increased the risk of NAFLD compared with mild severity of psoriasis (odds ratio = 2.25) (a large cohort study) [54]. Psoriasis complicated with NAFLD increased the risk of cardiovascular events (odds ratio = 6.0) (a small cohort study) [55]. Systemic therapy reduced the risk of psoriasis related NAFLD. Etanercept could be more efficacious to reduce the risk of developing hepatic fibrosis than PUVA therapy (a small cohort study) [56]. These findings suggest the importance of systemic therapy for severe psoriasis to impair the development of psoriasis-associated liver dysfunction.

Emerging evidence suggests a key contribution to NAFLD pathogenesis by Th17 cells. NAFLD model high-fat diet mice showed a higher frequency of IL-17 producing cells in the liver [57,58,59]. IL-17 neutralizing antibody treatment ameliorated liver damages [60]. NASH and subsequently HCC are prevented by IL-17A blocking antibodies [61]. IL-17-deficient mice were protected from NASH development [62].

## 6. Renal Dysfunction

Chronic kidney disease (CKD) is a syndrome defined as persistent alterations in kidney structure and function [63]. Nephrons are generated in weeks 12–36 of gestation in humans and new nephrons cannot be generated after this period. Severe kidney injury accelerates nephron loss, and glomerular hypertension increases nephron size as a compensatory action to maintain total the glomerular filtration rate and to reduce intraglomerular pressure. In addition, podocytes are necessary to become hypertrophied to maintain the filtration barrier along the enlarged filtration surface. Due to the limitation of podocyte hypertrophy, these alterations accelerate chronic kidney disease progression.

The link between psoriasis and renal dysfunction has been investigated by several clinical studies. A cohort study with 143,883 psoriasis patients showed that psoriasis was associated with the risk of chronic kidney disease (HR = 1.05) (a large cohort study) [64] and the severity of psoriasis was related to the increased risk of chronic kidney disease (mild psoriasis HR = 0.89; moderate psoriasis HR = 1.36; severe psoriasis HR = 1.584) (a large cohort study) [64]. Psoriasis (*n* = 199,808) was also associated with the risk of end-stage renal disease (HR = 1.34) (a large cohort study) [65]. The psoriatic arthritis group had a higher risk of chronic kidney disease (HR = 1.62, psoriasis patients *n* = 4344) (a large cohort study) [66] and end-stage renal disease (ESRD) (HR = 7.60, psoriasis patients *n* = 530,307) (a large cohort study) [67]. To evaluate the beneficial influence of psoriasis treatment for renal dysfunction, one study followed up on psoriasis patients (*n* = 92) to evaluate the continuous treatment of serum creatinine with biological therapy (a small cohort study) [68]. A decrease in serum creatinine was observed after 1 year of biological therapy. PASI was positively correlated with serum creatinine. These findings suggest that psoriasis treatment protects against the advancement of future renal dysfunction related to psoriasis inflammation.

Although the detailed mechanism of psoriasis-related renal dysfunction remains unclear, it has been speculated that psoriatic skin inflammatory response influences an inflammatory response in the kidney. Patients with ESRD showed that Th17 and Th2 cell frequencies were greater in the ESRD group [69]. The presence of IL-17A-expressing cells was positively related to the production of inflammatory cytokines and the degree of renal damage. IL-17A blockade ameliorated renal dysfunction [70]. An AT1R antagonist, losartan, significantly reduced the number of renal Th-17 cells and kidney fibrosis [71]. IL-17A-deficient mice were also protected from renal injury. These studies show that IL-17A contributes to the development of renal dysfunction [72].

## 7. Psychological Disorders

There are many different psychological disorders that are generally characterized by a combination of abnormal emotions and behaviors triggered by relationships with other people. Mental disorders such as depression and anxiety have highlighted the issue for clinicians because the burden of mental disorders continues to grow with significant impacts on health and society.

The association between psoriasis and the risk of psychiatric diseases has been evaluated by various studies. Psoriasis increased the risk of depression (HR = 1.18; psoriasis patients *n* = 10,868) (a large cohort study) [73], anxiety disorders (HR = 1.16; psoriasis patients *n* = 10,868) (a large cohort study) [73], somatoform disorder (HR = 1.21, psoriasis patients *n* = 10,868) (a large cohort study) [73]. The risks of depression were increased depending on the severity of psoriasis (mild psoriasis HR = 1.19; moderate psoriasis HR = 1.19; severe psoriasis HR = 1.50; psoriasis patients *n* = 247,755) (a large cohort study) [74]. Pediatrics PsA increased the risk of depression disorder, even in pediatric PsA patients (PsA/control incidence rate ratios = 2.38, pediatrics psoriasis patients *n* = 212) (a small cohort study) [75]. Psoriasis with depression was associated with the increased risk of craniocerebral vascular events, such as atrial fibrillation (psoriasis without depression HR = 1.32; psoriasis with depression HR = 1.74; psoriasis patients *n* = 56,496) (a large cohort study) [76]. Systemic therapy showed the potential to reduce the risk of psychiatric disease onset. Compared with conventional therapy or phototherapy, biologics decreased the risk for depressive symptoms (biologics HR = 0.76; phototherapy, HR = 1.05) (a large cohort study) [77].

A chronic, unpredictable, mild, stress-induced, depressive behavior model in a mouse showed that chronic stress upregulated the ratio of activated pro-inflammatory T helper 17 (Th17) in the liver and ileum [78]. This animal model also increased interleukin IL-17 and Anti-IL-17 treatment impaired anxiety and depression-like behavior [79]. These findings suggest that systemic psoriasis treatment might impair the risk of psychological disorders.

## 8. Hypothyroidism

Hypothyroidism is one of the most common pathological hormone deficiencies [80]. The variety of disease conditions and severities contribute to the development of hypothyroidism. The most common cause of acquired hypothyroidism is autoimmune thyroiditis. The role of autoimmunity in its pathogenesis is supported by the histological diffuse lymphocytic infiltration into the thyroid gland, the presence of thyroid autoantibodies circulation in almost all patients, and thyrocytes expressing the MHC class II proteins which is essential for activation of CD4 T cells specific for thyroid antigens [80].

Several studies have shown the relationship between hypothyroidism and psoriasis. The prevalence of autoimmune thyroiditis was higher in PsA patients than psoriasis (psoriasis *n* = 100 vs. PsA *n* = 108, 25.9% vs. 9.0%) (a small cohort study) [81]. These findings suggest possible pathogenesis of a psoriasis-associated inflammatory response involved in hypothyroidism.

As the mechanisms, Il-17 and IL-1β, seem to be associated with the development of hypothyroidism in patients with psoriasis, the frequency of Th17 cells in peripheral blood was significantly increased in patients with Hashimoto’s disease [82]. In addition, Tc17 cells also increased in Hashimoto disease patients [83]. The frequency of Th17 cells showed a positive correlation with the serum level of anti-thyroid peroxidase antibodies, anti-thyroglobulin antibodies, and TSH [84]. TSH and FT4 were negatively and positively correlated with IL-17 and IL-23, respectively [85]. The imbalanced immune condition of Th17 cells and Tregs were also observed in Hashimoto’s disease. Th17 cells enhanced the inflammatory response and effector cells which caused apoptosis and an inflammatory response in thyrocytes. Reduced Tregs also accelerated Th17 activity [86]. IL-1β was also involved in the pathogenesis of autoimmune thyroid disease. Th17 cells enhance differentiation and proliferation by IL-1β stimulation, which was higher in autoimmune thyroid disease patients [87]. Although there was no clinical study regarding the efficacy of psoriasis-associated inflammatory cytokine targeted inhibitor treatment, there was a single case report. A patient with juvenile idiopathic arthritis during adolescence complicated with type 1 diabetes mellitus and autoimmune Hashimoto’s thyroiditis received an anti-TNF drug, etanercept, which showed an improvement in thyroid function [88]. These findings suggest that the possible involvement of psoriatic cytokines mediated the amplification of inflammatory reaction in the thyroid which becomes a trigger to cause hypothyroidism.

## 9. Alzheimer’s Disease (AD)

AD is a common cause of acquired cognitive impairment in midlife and late life. AD is a neurodegenerative disorder caused by β-amyloid (Aβ)-containing extracellular plaques and tau-containing intracellular neurofibrillary tangles [89].

Various studies showed that psoriasis increased the risk of Alzheimer’s disease. A retrospective study showed an increased hazard ratio for AD. Psoriasis showed significant positive associations with AD (HR = 1.09, psoriasis patients *n* = 533,927) and the risk of AD was increased in psoriasis patients not receiving systemic therapy (HR = 1.10) (a large cohort study) [90]. Tumor Necrosis Factor (TNF) inhibitors decreased the risk of AD in patients with psoriasis [91]. Etanercept and adalimumab could impair the risk of AD in patients with psoriasis (Odds Ratio = 0.47, psoriasis patient *n* = 309,660) (a large cohort study). These findings indicate that psoriatic skin inflammation might become a trigger for the development of AD and cytokine targeted therapy might become a candidate to impair the progress of AD.

As mechanisms, Th17 cells were significantly increased in subjects with AD [92]. Amyloid β1-42 (Aβ1-42) injected into the hippocampus of rats induced AD [93] and this AD model showed Th17 cell infiltration into the brain [93]. In addition, increased interleukin (IL)-17 and IL-22 were observed in the hippocampus in both the cerebrospinal fluid and the serum [93]. Fas ligand was predominantly expressed by Th17 cells and was also related to neuronal apoptosis. [93] Furthermore, anti-IL-17 antibody treatment impaired Aβ1-42-induced neurodegeneration and pro-inflammatory cytokine production, and improved memory function [94]. Adalimumab also showed a protective effect mediated by decreased NF-κB activity which played an important role in neuroinflammatory transcription factors [95].

## 10. Chronic Rhinosinusitis

Chronic rhinosinusitis is inflammation of the mucosa of the nose and paranasal sinuses and is characterized by common symptoms including facial pain or pressure and nasal discharge [96]. The epithelium barrier and epithelial-mesenchymal transition to innate and adaptive immunity pathways are involved in the pathogenesis of chronic rhinosinusitis.

Several statistical analyses identified that the pre-existence of chronic rhinosinusitis also increased the future risk of psoriasis during a 5 year follow-up after the diagnosis of chronic rhinosinusitis (HR = 2.01, chronic rhinosinusitis patient *n* = 13.242) (a large cohort study) [97]. IL-17 expression was increased in patients with chronic rhinosinusitis with nasal polyps [98,99,100,101]. Primary human nasal epithelial cells taken from chronic rhinosinusitis with nasal polyp patients showed that the Th17 cytokines IL-17, IL-22, and IL-26 enhanced the significant disruption of the epithelial barrier, evidenced by a loss of transepithelial electrical resistance and increased paracellular permeability [102]. On the contrary, IL-17 and TGF-β1 increased Th2-related cytokine production [103].

## 11. Endometriosis

Endometriosis is a common chronic inflammatory disease in women in which tissue resembling the endometrium is observed outside the uterus, mainly in the pelvic area [104]. Epithelial-mesenchymal transition and alteration of immune profiles and inflammatory responses are also involved in the pathogenesis of endometriosis. Interestingly, a statistical study identified a higher prevalence rate of autoimmune diseases, such as SLE and rheumatoid arthritis, in women with endometriosis [105].

The correlation between endometriosis and psoriasis has been reported. A history of psoriasis with concomitant PsA was related to a higher risk of endometriosis (HR = 2.01, psoriasis patient *n* = 110) (a small cohort study) [106]. Although the mechanism of psoriasis-associated endometriosis remains unclear, the potential importance of Th17 cells is thought to be associated with the development of endometriosis. Immunohistochemistry revealed the localization of IL-17A+ cells were observed in the tissues taken from endometriosis. IL-17A enhanced angiogenesis and the production of inflammatory cytokines and chemokines [107], possibly enhancing inflammatory reaction in endometriosis.

## 12. Uveitis

Uveitis is an inflammatory process of the portion in the eye uvea, which is composed of the choroid, ciliary body, and iris [108]. Uveitis is most often idiopathic and has been related to traumatic, inflammatory, and infectious processes. Systemic inflammatory disorders are closely related to anterior uveitis such as inflammatory bowel disease, sarcoidosis, and Behcet’s disease.

Eye inflammation was closely associated with the presence of autoimmune diseases [109]. Mild or severe psoriasis, or psoriatic arthritis, increased the uveitis risk (mild psoriasis HR = 1.38, severe psoriasis HR = 1.40, and PsA HR = 2.50, psoriasis patient *n* = 74,129) (a large cohort study) [110]. A model of a uveitis animal experiment showed IL-17 was elevated in an experimental autoimmune uveitis model mouse and TNF-α was upregulated in retinal cells [111]. IL-6 deficient mice and IL-23 deficient mice impaired Th17 cell-mediated autoimmune uveitis [112]. Oral vitamin D analog calcitriol prevented the development of uveitis as well as IL-17 production [113].

## 13. Gut Inflammation

The gut immune system interacts with skin immunity. A high frequency of inflammatory lesions in the gut was seen in 16% of psoriasis patients (a small cohort study) [114]. Among these patients, HLA-B27 and Bw62 showed a higher prevalence of gut inflammation in 60 and 50% of patients with psoriasis [114]. IL-17 is closely associated with gut inflammation. The adoptive transfer of IL-17A- or IL-17F-deficient T lymphocytes into T-cell deficient mice caused severe colitis [115]. In the gut, IL-21 plays an essential role in Th17 differentiation. IL-21-deficient mice or IL-21 neutralizing antibody treatments could not induce Th17 differentiation [116]. These findings suggest that Th17 plays a central role as a positive driver for the induction of gut inflammation. In accordance with other inflammatory gut diseases, Th17 is also involved in the pathogenesis of Crohn’s disease [117], which also accompanies psoriasis [118]; therefore, these findings suggest that Th17 is involved in the development of psoriasis and gut inflammation.

## 14. Chronic Obstructive Pulmonary Disease (COPD)

There are several studies to show the influence of psoriasis on the risk of COPD. A case-control study that included 12,502 patients with psoriasis showed the high prevalence of COPD in patients with psoriasis by multivariate logistic regression analysis (OR 1.27) (a large cohort study) [119]. Another study also showed an increased risk of COPD in psoriasis patients (*n* = 2095) (HR = 2.35) (a large cohort study) [120]. In particular, the risk of COPD was increased in males (HR = 2.38) and people over 50 years old (HR = 2.19) (a large cohort study) [120]. The severity of psoriasis determined the risk of COPD. A study showed that the risk of COPD was high in patients with psoriasis (*n* = 13,418) (odds ratio = 1.90) and severe psoriasis caused a higher risk of COPD (odds ratio = 2.15) (a large cohort study) [121]; however, the actual interaction of psoriatic skin inflammation with COPD remains unclear. Th17 and Tc17 are involved in the development of COPD [122]. The cigarette smoking-induced COPD model showed increased key cytokines in psoriasis patients, such as TNF-α and IL-17 [123]. Increased IL-17 enhances the expression of pattern recognition receptors, such as TLR3 and TLR7 [124], leading to a high sensitivity inflammatory reaction to the pathogens.

## 15. Whole Interaction of Psoriasis to Systemic Organ Inflammation

**Figure 2:**
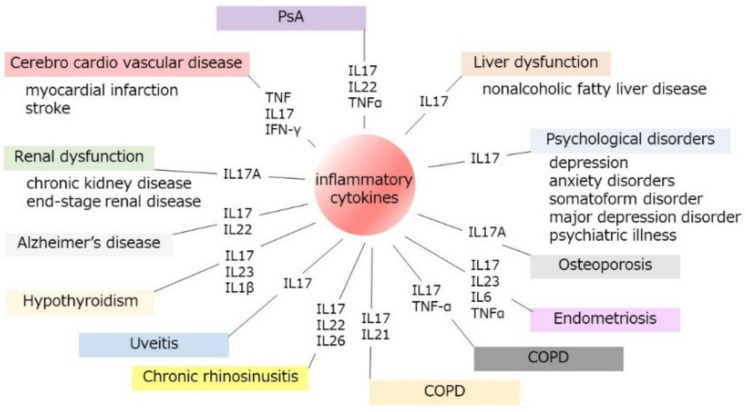
Interactions of psoriatic inflammation to systemic inflammatory disorders. Psoriatic inflammation is involved in the development of systemic organ dysfunctions. As skin occupies a large surface field in the human body, the characteristics of psoriasis as large surface involvement influences the extension of abundant inflammatory involvement in systemic inflammatory disorders.

We summarized the whole interaction of psoriatic inflammation to other organs in Figure 2 and Table 1. As skin occupies a large surface field in the human body, the characteristics of psoriasis as large surface involvement influence the extension of abundant inflammatory involvement in systemic inflammatory disorders. Consistently, systemic therapy, especially biologics, can impair the development of these systemic inflammatory reactions and decrease the development of the risk of other inflammatory organ dysfunctions, which indicates the importance of regulating the cutaneous inflammatory response in psoriasis patients. On the contrary, other organ-related inflammations are known to become risks for psoriasis. For instance, the history of uveitis increased the future risk of psoriasis [110], suggesting that immunological interactions between organs orchestrate the extent to which another inflammatory site leads to the dysfunction of other systemic diseases; therefore, the common key factors in the pathogenesis of systemic inflammatory diseases may be highlighted in the future direction of the importance of systemic therapy.

## 16. Conclusions

Psoriasis skin inflammation influences various systemic inflammatory disorders to exacerbate inflammatory reactions and the development of inflammatory diseases. Almost all secondary inflammatory sites depend on the severity of psoriasis inflammation and systemic treatment, especially biologics, which are useful to prevent systemic inflammation reactions; therefore, it is an important issue for clinicians to evaluate the accurate inflammation degree and severity, in order to determine the therapeutic option. In addition, the knowledge gained from this review provides the necessary evaluation of systemic organ influences before the administration of systemic therapy to determine the appropriate therapeutic option.

A strong association was observed in cerebrocardiovascular diseases, osteoporosis, NAFLD, end-stage renal disease, chronic rhinosinusitis, endometriosis, and COPD as shown in Table 1; however, as a limitation of this review, there were several small cohort studies on hypothyroidism, endometriosis, and gut inflammation; therefore, the risk of these inflammatory diseases associated with psoriasis should be determined by further large cohort studies.

One of the problems concerns which kinds of therapeutic options are better to regulate the development of systemic inflammatory disorders. In the underlying mechanism of psoriasis, TNF-α or IL-23 are the upper stream cytokines, whereas IL-17 are the downstream cytokines. Various biologic options are currently available and these systemic organ influences will be determined by further clinical trials. As psoriasis skin inflammation is a strong inflammatory trigger for systemic inflammatory disorders, there might be unknown organ influences; therefore, further investigation to elucidate unknown systemic influences should be clarified by further investigation.

## Figures and Tables

**Table 1:** The clinical evidence of psoriasis and other inflammatory diseases.

Inflammatory Diseases	The Influence of Psoriasis
Cerebrocardiovascular diseases	High risk [7,30,31,32,33]
Osteoporosis	High risk [8,46,47]
NAFLD	High risk [52,53,54,55]
Renal dysfunction	
Chronic kidney disease	Moderate risk [64,66]
End stage renal disease	High risk [65,67]
Psychological disorders	
Depression	Moderate risk [73,76]
Anxiety disorder	Moderate risk [73]
Somatoform disorder	Moderate risk [73]
Alzheimer’ disease	Moderate risk [90]
Chronic rhinosinusitis	High risk [97]
Endometriosis	High risk [106]
Uveitis	Moderate risk [110]
COPD	High risk [119,120,121]

Graded high risk and moderate risk is less than two in the hazard ratio, odds ratio, or rate ratio, respectively.

## Data Availability

Not applicable.
